# Increased IL-21 Expression Induces Granzyme B in Peripheral CD5^+^ B Cells as a Potential Counter-Regulatory Effect in Primary Sjögren's Syndrome

**DOI:** 10.1155/2016/4328372

**Published:** 2016-01-18

**Authors:** Gábor Papp, Edit Gyimesi, Krisztina Szabó, Éva Zöld, Margit Zeher

**Affiliations:** Division of Clinical Immunology, Faculty of Medicine, University of Debrecen, Móricz Zsigmond Street 22, Debrecen 4032, Hungary

## Abstract

Recently, we reported elevated proportions of circulating follicular T helper cells and higher levels of interleukin- (IL-) 21 in primary Sjögren's syndrome (pSS). Interaction of invariant natural killer T (iNKT) cells with B cells and granzyme B (GrB) production may be also important in pSS. Thirty-two pSS patients and 24 healthy controls were enrolled in our study. We investigated the expression of intracellular GrB and IL-21 receptor (IL-21R) of CD19^+^CD5^+^ and CD19^+^CD5^−^ B cells; furthermore, we determined the IL-21 expression of iNKT cells as well. We also assessed the proportion of transitional (CD19^+^CD24^high^CD38^high^), mature (CD19^+^CD24^int^CD38^int^) and primarily memory (CD19^+^CD24^high^CD38^−^) B cells. CD5^+^ but not CD5^−^ B cells showed elevated GrB and IL-21R expression in pSS; additionally IL-21 expression of iNKT cells was also elevated. The ratios of transitional and mature B cells were elevated in pSS, while primarily memory B cell percentages were decreased, which correlated with GrB and IL-21R expression of CD19^+^ B cells. Our results suggest that enhanced IL-21R expression of CD19^+^CD5^+^ B cells and production of IL-21 by iNKT cells may play an important role in the pathogenesis of pSS by regulating CD19^+^CD5^+^ B cell functions and increasing GrB production, presumably leading to a counter-regulatory effect in the disease.

## 1. Introduction

Primary Sjögren's syndrome (pSS) is a common systemic autoimmune disease characterized by inflammation and consequential destruction of exocrine glands. In the last decades, large amount of studies confirmed that B cell activation plays a crucial role in the pathogenesis of pSS through antigen presentation, autoantibody production, and secretion of several proinflammatory factors. The pathological hallmark of pSS is extensive lymphocytic infiltration in salivary glands. The extension and structural arrangement of the infiltrations vary between wide limits; even ectopic germinal centres may develop. These structures are characterized by* in situ* autoantibody production and high expression of homing and retentive chemokines and adhesion molecules. It was reported that the number of ectopic GCs in salivary glands correlates with the severity of inflammation and anti-SSA/SSB autoantibody production [1, 2]. Additionally, formation of ectopic GCs in glandular tissues carries a higher risk of developing B cell lymphoma in pSS [3].

The proliferation and differentiation of B cells in GCs highly depend on their collaboration with follicular helper T (T_FH_) cells, which are generated from peripheral naive CD4^+^ T cells in the T cell zone of lymphoid organs. The appropriate interaction of activated B cells and T_FH_ cells is crucial for the generation of extrafollicular short-lived low-affinity plasma cells, high-affinity memory B cells, and long-lived plasma cells [4]. Recent investigations shed light on altered T_FH_ profiles in various autoimmune conditions, suggesting the important role of T_FH_ cells and IL-21 cytokine secretion in autoreactive B cell activation and autoantibody production [4]. In labial salivary gland biopsies of pSS patients, T_FH_ cell markers (CD84, PD-1, and Bcl-6) were detected in the lymphocytic infiltrations, especially, in more organized lymphoid structures [5]. In peripheral blood, elevated CD4^+^CXCR5^+^ICOS^+^PD-1^+^T_FH_-like cell percentages were reported in pSS, which showed strong association with anti-SSA and/or anti-SSB autoantibody positivity. Moreover, patients with higher T_FH_ cell proportions had elevated IL-21 serum levels [6].

As part of immune responses, IL-21 is produced by activated CD4^+^ T cells and natural killer (NK) T cells. IL-21-activated B cells produce IL-6, which further activates B cells in an autocrine manner and contributes to T_FH_ cell differentiation and autoantibody production [7]. Recently, Lindner et al. revealed that IL-21 induces B cells to produce and secrete the active form of the cytotoxic serine protease granzyme B (GrB) and gain immune regulatory properties by limiting T cell proliferation by a GrB-dependent degradation of T cell receptor *ζ*-chain [8]. In systemic lupus erythematosus, CD19^+^CD5^+^ B cells were reported to be one of the main sources of GrB [9]. These observations raise the possibility that beside the key role of T_FH_ cells in B cell activation, the interaction of IL-21-producing invariant NKT (iNKT) cells with B cells and GrB production may also play a potential role in the pathogenesis of pSS.

In our study, we investigated the expression of IL-21 receptor (IL-21R) and GrB of B cell subsets; we assessed the IL-21 expression of iNKT cells and determined the distribution of transitional, mature, and primarily memory B cell subsets in the peripheral blood of pSS patients.

## 2. Materials and Methods

### 2.1. Patients and Healthy Individuals

We enrolled 32 pSS patients in the study, who visited our outpatient clinic for systemic autoimmune diseases in the Division of Clinical Immunology of the University of Debrecen for a regular medical check-up between April and June 2014. Exclusion criteria included other autoimmune, allergic, and malignant diseases, pregnancy, immunosuppressive or immunomodulating medications, and ongoing infections. The diagnosis was based on the European-American consensus criteria [10]. Twenty-four age- and sex-matched healthy individuals served as controls.

Among pSS patients, 18 suffered from extraglandular manifestations (EGMs), while 14 had glandular symptoms only. The distribution of EGMs of pSS patients was as follows: polyarthralgia/polyarthritis *n* = 16, Raynaud's phenomenon *n* = 7, polyneuropathies *n* = 4, and vasculitis *n* = 3. Vasculitis or other EGMs needing immunosuppressive treatment were newly recognised or in inactive status. Informed written consent was obtained from the subjects, and the study has been approved by the Ethics Committee of the University of Debrecen. All experiments carried out were in compliance with the Declaration of Helsinki. Data on subjects enrolled in the study are summarized in Table 1.

### 2.2. Analysis of GrB Production and IL-21R Expression of CD19^+^CD5^+^ and CD19^+^CD5^−^ B Cells

For the evaluation of intracellular GrB production of CD19^+^CD5^+^ and CD19^+^CD5^−^ B cell, peripheral blood mononuclear cells (PBMCs) were isolated from heparinized venous blood sample by Ficoll-Histopaque (Sigma-Aldrich, St. Louis, MO, USA) density-gradient centrifugation. Cells were then harvested and washed twice and cultured in modified RPMI 1640 medium with GLUTAMAX-I (Life Technologies Corporation, Carlsbad, CA, USA) supplemented with 100 U/mL penicillin, 100 ng/mL streptomycin, and 10% heat-inactivated fetal calf serum (FCS) (Life Technologies). Cells were stimulated at 1 × 10^6^ cells/mL concentration using 6,5 *μ*g/mL anti-human B cell receptor (BCR) antibody (Jackson ImmunoResearch, West Grove, PA, USA) and 50 ng/mL recombinant human IL-21 (R&D Systems, Inc., Minneapolis, MN, USA) for 18 hours at 37°C in an atmosphere containing 5% CO_2_, based on the protocol described previously by Hagn et al. [9]. The transport of* de novo* synthesized cytokines from the Golgi apparatus was inhibited by 10 *μ*g/mL brefeldin-A (Sigma-Aldrich). Unstimulated cells served as controls. Next, cells were stained with fluorescein isothiocyanate- (FITC-) conjugated anti-CD5 and phycoerythrin-cyanine (PC)5-labelled anti-CD19 antibodies (BD Biosciences, San Diego, CA, USA, and Beckman Coulter Inc., Miami, FL, USA) for 30 min at 4°C. The cells then were fixed and permeabilized with Intraprep permeabilization reagent (Beckman Coulter) according to the manufacturer's instructions, and intracellular GrB were stained with phycoerythrin- (PE-) conjugated anti-GrB antibody (BD Biosciences).

For the evaluation of IL-21R expression, anti-CD5-FITC, anti-CD19-PC5, and anti-IL-21R- (CD360-) PE antibodies were used (BD Biosciences, Beckman Coulter and BioLegend, San Diego, CA, USA), and mean fluorescence intensity (MFI) was determined. Mouse immunoglobulin (Ig) G1 antibodies were used as isotype control throughout the experiments. The measurements were performed on a FACSCalibur flow cytometer (Becton Dickinson, Franklin Lakes, NJ, USA). CD19^+^, CD19^+^CD5^+^, and CD19^+^CD5^−^ B cells were quantified as their percentage in the entire lymphocyte population. GrB positivity and IL-21R expression levels were quantified within the investigated B cell subpopulations.

### 2.3. Assessment of Peripheral iNKT Cell Percentages and Their IL-21 Expression

In order to determine iNKT cells from heparinized blood samples, the following monoclonal antibodies to cell surface markers were used: FITC-conjugated anti-CD3 and PE-conjugated 6B11 (BD Biosciences). Samples were processed according to the Coulter Q-PREP protocol and system (Beckman Coulter Inc.). Briefly, cells from 50 *μ*L of whole blood were stained with 10 *μ*L of each monoclonal antibody. After 20 min incubation, red blood cells were haemolysed and leucocytes were washed in phosphate-buffered saline (PBS) supplemented with bovine serum albumin (BSA) (10 mg/L) and sodium-azide (2 mg/L). The cells were fixed subsequently using 500 *μ*L of 1% paraformaldehyde.

The cytoplasmic IL-21 content of circulating iNKT cells was also determined by flow cytometry. Briefly, isolated PBMCs were cultured at a concentration of 2 × 10^6^/mL in modified RPMI 1640 medium with GLUTAMAX-I (Life Technologies) supplemented with 100 U/mL penicillin, 100 ng/mL streptomycin, and 10% heat-inactivated fetal calf serum (Life Technologies). Cells were stimulated using 25 ng/mL phorbol-myristate-acetate (Sigma Aldrich) and 1 *μ*g/mL ionomycin (Sigma Aldrich) for 6 hours at 37°C in an atmosphere containing 5% CO_2_. The transport of* de novo* synthesized cytokines from the Golgi apparatus was inhibited by 10 *μ*g/mL brefeldin-A (Sigma Aldrich). After stimulation, cells were stained with anti-CD3-FITC and 6B11-PE antibodies (BD Biosciences) for 30 min at 4°C. The cells then were fixed and permeabilized with Intraprep permeabilization reagent (Beckman Coulter Inc.) according to the manufacturer's instructions, and intracellular cytokines were stained with allophycocyanin- (APC-) conjugated anti-IL-21 antibody (BioLegend). Mouse IgG1 antibodies were used as isotype control throughout the experiments. The measurements were performed on a FACSCalibur flow cytometer (Becton Dickinson). The CD3^+^6B11^+^ iNKT cells were quantified as their percentage in the entire lymphocyte population, while IL-21^+^ iNKT cells were determined as their proportions in the population of CD3^+^6B11^+^ iNKT cells.

### 2.4. Determination of Transitional, Mature, and Primarily Memory B Cells

PBMCs were isolated from heparinized venous blood sample by Ficoll-Histopaque (Sigma-Aldrich) density-gradient centrifugation. Cells were then harvested and washed twice and stained for 20 min at 4°C using specific antibodies. To identify transitional, mature, and primarily memory B cell subpopulations, cells were stained with the combination of the following monoclonal antibodies: PC5-labelled anti-CD19 (Beckman Coulter), Alexa Fluor 488-labelled anti-CD24 (BioLegend), and PC7-labelled anti-CD38 (BD Biosciences). After 30 min incubation, leucocytes were washed in phosphate-buffered saline (PBS) supplemented with bovine serum albumin (BSA) (10 g/L) and sodium-azide (2 mg/L). The cells were fixed subsequently using 500 *μ*L of 1% paraformaldehyde. Mouse immunoglobulin (Ig) G1 antibodies were used as isotype control throughout the experiments. Samples were processed according to the Coulter Q-PREP protocol and system (Beckman Coulter Inc., Miami, FL, USA). Measurements were performed on a Coulter FC500 flow cytometer (Beckman Coulter Inc.). CD19^+^CD24^high^CD38^high^ (transitional), CD19^+^CD24^int^CD38^int^ (mature), and CD19^+^CD24^high^CD38^−^ (primarily memory) B cells were quantified as their percentage in the CD19^+^ lymphocyte population.

### 2.5. Assessment of Anti-dsDNA, Anti-Ro/SSA, and Anti-La/SSB Autoantibodies

Autoantibodies were determined by enzyme-linked immunosorbent assay (ELISA) technique with AUTOSTAT II kits (Hycor Biomedical, Indianapolis, IN, USA), according to the manufacturer's instructions.

### 2.6. Statistical Analysis

The SPSS ver. 20.0 (SPSS Inc., Chicago, IL, UDA) was used for statistical analysis. To assess the distribution of the data Kolmogorov-Smirnov test was used. In cases of normal distribution, we determined mean ± standard deviation (SD) values and used two-sample *t*-test for statistical evaluation of the experimental data. In cases of distributions different from normal, median, minimum, and maximum values were calculated, and the Mann-Whitney *U* test was used. When the strength of the linear relationship between two variables was evaluated, Pearson's correlation coefficient was used, while in cases of nonnormal distribution, Spearman's correlation coefficient was applied. Differences were considered statistically significant at *p* < 0.05.

## 3. Results

### 3.1. Granzyme B Production Capability of B Cells Is Increased in pSS

**Figure 1:**
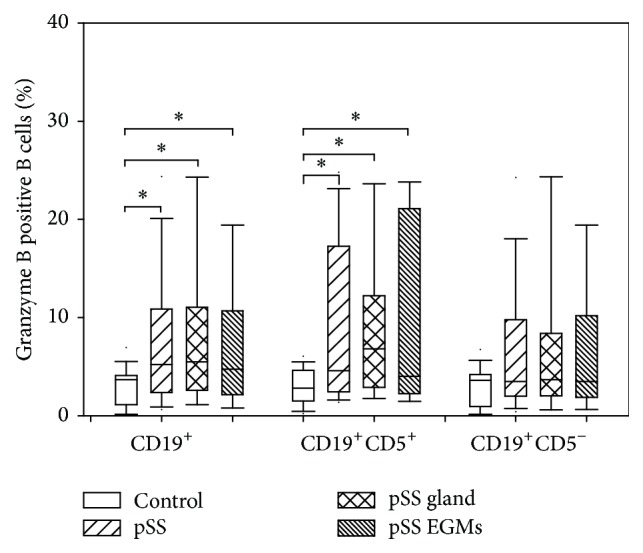
Granzyme B expression of B cell subsets. Box plots show median, upper, and lower quartiles; lines extending vertically from boxes indicate 5–95 percentile ranges. Statistically significant differences are indicated by *∗*.

We found no significant differences in peripheral blood CD19^+^, CD19^+^CD5^+^, and CD19^+^CD5^−^ B cell percentages between pSS patients, subgroups of patients, and healthy controls (data not shown). When GrB expressions of these B cell subsets were investigated, in the whole CD19^+^ B cell population, significantly enhanced GrB expression was observed in the whole group of pSS patients (median (min–max): 5.225 (0.41–28.1)% versus 3.69 (0.04–7.29)%, resp., *p* = 0.0121) and in all subgroups of patients (pSS glandular: median (min–max): 5.505 (0.41–28.1)% versus 3.69 (0.04–7.29)%, resp., *p* = 0.0261; pSS EGMs: median (min–max): 4.735 (0.73–22.34)% versus 3.69 (0.04–7.29)%, resp., *p* = 0.0486), compared to the values measured in the healthy controls (Figure 1).

When we divided CD19^+^ B cell population on the basis of their CD5 positivity, we found that only CD19^+^CD5^+^ B cells expressed GrB significantly higher in each group of pSS patients, compared to control values (pSS: median (min–max): 4.59 (1.04–26.45)% versus 2.8 (0.18–6.13)%, resp., *p* = 0.0086; pSS glandular: median (min–max): 6.81 (1.6–26.45)% versus 2.8 (0.18–6.13)%, resp., *p* = 0.0101; pSS EGMs: median (min–max): 4.03 (1.04–23.91)% versus 2.8 (0.18–6.13)%, resp., *p* = 0.0354) (Figure 1).

### 3.2. IL-21 Receptor Expression Is Enhanced on CD19^+^CD5^+^ B Cells in pSS

**Figure 2:**
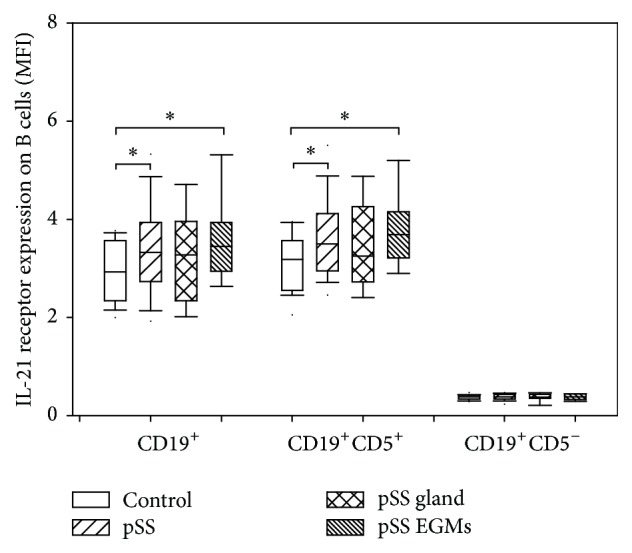
Interleukin-21 receptor expression of B cell subsets. Box plots show median, upper, and lower quartiles; lines extending vertically from boxes indicate 5–95 percentile ranges. Statistically significant differences are indicated by *∗*.

We observed significantly enhanced IL-21R expression on CD19^+^ B cells in the whole group of pSS patients (mean ± SD: 3.434 ± 0.929 MFI versus 2.961 ± 0.585 MFI, resp., *p* = 0.0288) and in subgroup of patients with EGMs, compared to values measured in healthy individuals (mean ± SD: 3.578 ± 0.901 MFI versus 2.961 ± 0.585 MFI, resp., *p* = 0.0195) (Figure 2).

When we divided CD19^+^ B cell population on the basis of their CD5 positivity, we found that only CD19^+^CD5^+^ B cells expressed IL-21R significantly higher in the whole group of pSS patients (mean ± SD: 3.677 ± 0.894 MFI versus 3.145 ± 0.564 MFI, resp., *p* = 0.0114) and in the subgroup of patients with EGMs, compared to control values (mean ± SD: 3.834 ± 0.872 MFI versus 3.145 ± 0.564 MFI, resp., *p* = 0.0078) (Figure 2).

**Figure 3:**
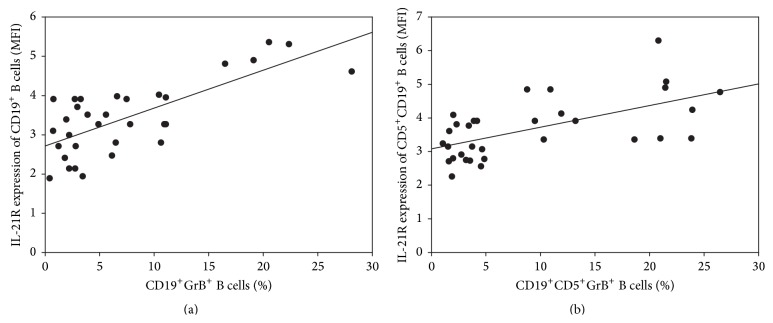
Correlation analysis between granzyme B and interleukin-21 receptor expression in (a) CD19^+^ B cells (*R* = 0.6261, *p* = 0.0001) and (b) CD19^+^CD5^+^ B cells (*R* = 0.5949, *p* = 0.0003).

Moreover, we found positive correlation between the percentages of GrB positivity and the level of IL-21R expression within both CD19^+^ B cells (*R* = 0.6261, *p* = 0.0001) and CD19^+^CD5^+^ B cells (*R* = 0.5949, *p* = 0.0003) in pSS (Figures 3(a) and 3(b)).

### 3.3. Elevated Intracellular IL-21 Cytokine Expression of iNKT Cells in pSS Patients

We observed no significant differences in the peripheral blood iNKT percentages between pSS patients and controls (data not shown). However, in the pSS patient population, we found significantly elevated percentages of IL-21-producing iNKT cells within iNKT population, compared to the values determined in healthy individuals (median (min–max): 2.81 (0.1–29.02)% versus 1.16 (0.1–12.1)%, resp., *p* = 0.0141).

### 3.4. Transitional, Mature, and Primarily Memory B Cell Proportions and Presence of Autoantibodies in pSS

**Figure 4:**
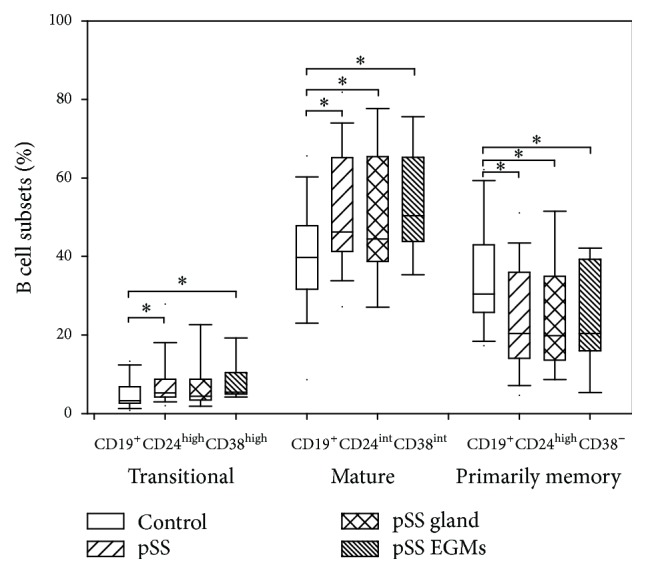
Percentages of transitional, mature, and primarily memory B cells. Box plots show median, upper, and lower quartiles; lines extending vertically from boxes indicate 5–95 percentile ranges. Statistically significant differences are indicated by *∗*.

The distribution of B cells was also evaluated in the study (Figure 4). The percentages of CD19^+^CD24^high^CD38^high^ transitional B cells were significantly higher in pSS patients compared to healthy control (median (min–max): 5.21 (1.68–27.83)% versus 3.19 (0.57–13.4)%, resp., *p* = 0.0079). Regarding the subgroups of patients, subjects suffering from EGMs had also elevated percentages (median (min–max): 5.44 (3.41–27.83)% versus 3.19 (0.57–13.4)%, resp., *p* = 0.0038), while values of patients with glandular symptoms did not differ significantly from the results of healthy individuals.

The proportions of CD19^+^CD24^int^CD38^int^ mature B cells were significantly elevated in the whole group of pSS patients (mean ± SD: 52.241 ± 15.245% versus 39.628 ± 13.958%, resp., *p* = 0.0021) and in both of pSS subgroups (pSS glandular: mean ± SD: 50.885 ± 17.618% versus 39.628 ± 13.958%, resp., *p* = 0.0343; pSS EGMs: mean ± SD: 53.296 ± 13.555% versus 39.628 ± 13.958%, resp., *p* = 0.0026), compared to control values.

On the contrary, the frequency of CD19^+^CD24^high^CD38^−^ primarily memory B cells were significantly decreased in all groups of pSS patients, compared to controls (pSS: mean ± SD: 24.166 ± 13.712% versus 35.083 ± 13.887%, resp., *p* = 0.0046; pSS gland: mean ± SD: 23.798 ± 14.422% versus 35.083 ± 13.887%, resp., *p* = 0.0214; pSS EGMs: mean ± SD: 24.452 ± 13.551% versus 35.083 ± 13.887%, resp., *p* = 0.0165).

**Figure 5:**
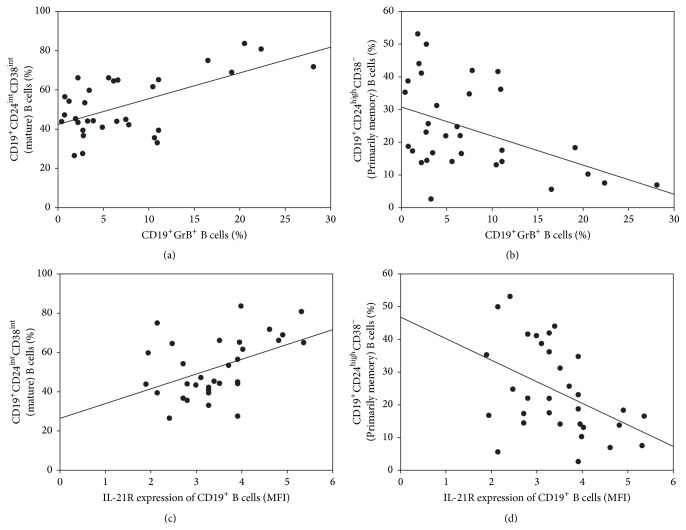
Correlation analysis between granzyme B and interleukin-21 receptor expression and B cell subsets. Associations between granzyme B expression of CD19^+^ B cells and (a) percentages of CD19^+^CD24^int^CD38^int^ mature B cells (*R* = 0.4022, *p* = 0.0224) as well as (b) percentages of CD19^+^CD24^high^CD38^−^ primarily memory B cells (*R* = −0.4565, *p* = 0.0086). Correlations between interleukin-21 receptor expression of CD19^+^ B cells and (c) percentages of CD19^+^CD24^int^CD38^int^ mature B cells (*R* = 0.4935, *p* = 0.0041) as well as (d) percentages of CD19^+^CD24^high^CD38^−^ primarily memory B cells (*R* = −0.4696, *p* = 0.0067).

Additionally, we found a positive correlation between the percentages of GrB positive CD19^+^ B cells and mature B cells (*R* = 0.4022, *p* = 0.0224), while a negative correlation between the percentages of GrB positive CD19^+^ B cells and primarily memory B cells (*R* = −0.4565, *p* = 0.0086) (Figures 5(a) and 5(b)). Similarly, positive correlation was observed between the levels of IL-21R expression of CD19^+^ B cells (*R* = 0.4935, *p* = 0.0041), while negative correlation was revealed between the levels of IL-21R expression of CD19^+^ B cells and primarily memory B cells (*R* = −0.4696, *p* = 0.0067) (Figures 5(c) and 5(d)). We observed no further correlations between the other investigated parameters.

Thirteen individuals were positive for anti-Ro/SSA and/or anti-La/SSB antibody among pSS patients. We observed no association between the presence of autoantibodies and the investigated parameters.

## 4. Discussion

Formerly, we reported higher level of circulating IL-21 in pSS and found increased circulating T_FH_ cell percentages in the disease [6]. In the present study, we observed elevated IL-21 expression of iNKT cells, which, along with the increased IL-21R expression of B cells, may contribute to the enhanced B cell activation in pSS. Interestingly, B cells expressing enhanced IL-21R on surface were typically positive for surface antigen CD5. Furthermore, when we divided patients into two groups based on the presence or absence of EGMs, significant increase in expression of IL-21R was found only in the group of EGM patients, compared to the control values. This observation is in line with our previous results; namely, significantly elevated ratio of peripheral T_FH_ cells was observed only in pSS patients with EGMs and not in those without EGMs; furthermore, higher IL-21 cytokine concentrations were associated with the presence of EGMs [6]. Regarding the IL-21 overproduction of iNKT cells, we did not find marked difference between the subgroups of patients.

**Figure 6:**
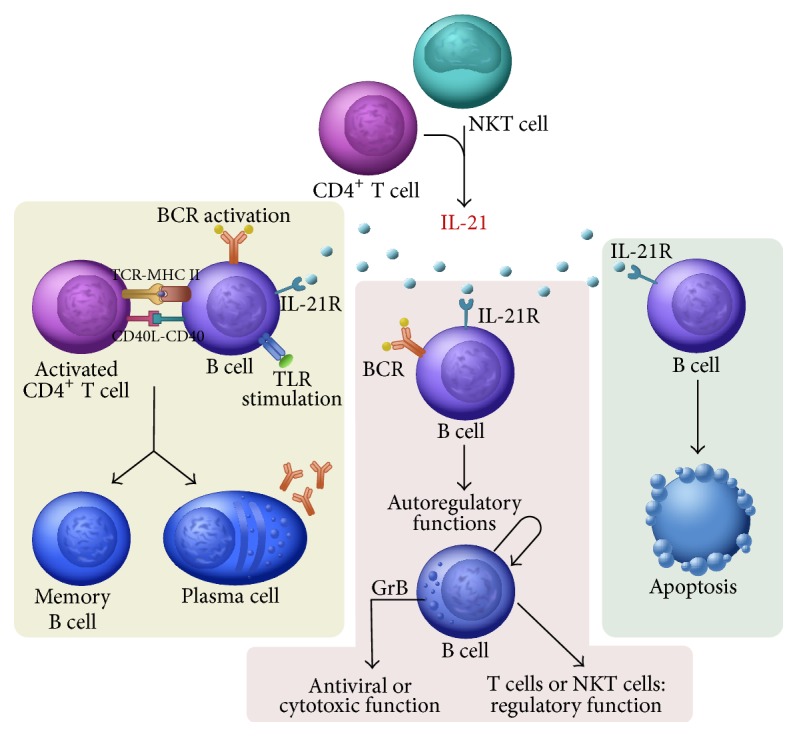
Possible results of interlukin-21 (IL-21) stimulation on B cells. In the presence of CD40 ligation and either a B cell receptor (BCR) or a Toll-like receptor signal, IL-21 stimulation fundamentally promotes B cell activation and differentiation into memory and plasma cells [6]. The combination of IL-21 stimulation and BCR engagement in the absence of CD40 ligation enables B cells to produce and secrete active form of granzyme B (GrB). GrB-producing B cells may acquire antiviral immune function and/or a regulatory function on themselves or other immune cells including T and NKT cells [15]. In the absence of both BCR engagement and T cell help, IL-21 stimulation induces B cell apoptosis [11].

Basically, IL-21 stimulation on B cells may have at least three outcomes. In the absence of both B cell receptor (BCR) engagement and T cell help, it induces B cell apoptosis [11]. In the presence of CD40 ligation and either a BCR or a Toll-like receptor signal, IL-21 stimulation fundamentally promotes B cell activation and differentiation processes into memory and plasma cells. However, the combination of IL-21 stimulation and BCR engagement in the absence of CD40 ligation enables B cells to produce and secrete the active form of GrB [12] (Figure 6). Besides its important and well-known function in antiviral immune responses, GrB secretion plays a potential role in the regulation of autoimmune responses as well, through different mechanisms. The spectrum of functions exhibited by GrB includes antigen processing, matrix degradation, cleavage, and activation of inflammatory cytokines and immunoregulatory effects [13, 14]. Consequently, GrB-producing B cells may acquire a regulatory function and effects on other immune cells; furthermore, leakage of GrB in the cytoplasm may also result in the apoptotic demise of autoreactive B cells [15]. Based on our observations, GrB expression of B cells, especially in CD19^+^CD5^+^ ones, is significantly increased in pSS regardless of the presence of EGMs. Importantly, we observed a clear positive correlation between GrB production and IL-21R expression on CD19^+^ B and CD19^+^CD5^+^ B cells as well. Additionally, we also found further associations with the altered distribution of transitional, mature, and primarily memory B cells. Our observations, namely, elevated mature B cell proportions and decreased primarily memory B cell percentages, are in line with former observations [16] pointing at the hyperactivated state of B cells in pSS patients. In our study, we revealed that not only IL-21R expression but also GrB production on CD19^+^ B cells correlates positively with the frequency of CD19^+^CD24^high^CD38^high^ transitional and CD19^+^CD24^int^CD38^int^ mature B cell subsets and correlates negatively with CD19^+^CD24^high^CD38^−^ primarily memory B cells.

## 5. Conclusions

Based on our results, we assume that, parallel with the pathologic B cell activation and enhanced autoantibody production driven by the expansion of T_FH_ cells and their pronounced IL-21 expression, GrB production is mainly induced in CD5^+^ B cell subsets in pSS, which could be part of an increased counter-regulatory reaction, presumably compensating the derailed, disproportional immune responses. We believe that further investigation on the activation and function of GrB-producing regulatory B cells will open new avenues to understand the B cell operation and autoimmune processes in pSS; furthermore, the modulation of these cells could be a potentially powerful element of the novel therapeutic selection in the disease.

## Figures and Tables

**Table 1:** The demographic characteristics of subjects enrolled in the study.

	Healthy individuals	pSS patients	Patients with EGMs	Patients without EGMs
Number	24	32	18	14
Age (years), mean ± SD	53.69 ± 9.12	58.98 ± 7.67	60.48 ± 6.47	57.04 ± 9.12
Sex (man/woman)	2/22	2/30	1/17	1/13

EGMs: extraglandular manifestations; pSS: primary Sjögren's syndrome.

## References

[1] Ramos-Casals M., Brito-Zerón P., Sisó-Almirall A., Bosch X. (2012). Primary Sjögren syndrome. *British Medical Journal*.

[2] Zeher M., Zeher M., Szodoray P. (2009). Sjögren's syndrome. *Sjögren's Syndrome and Associated Disorder*.

[3] Illes A., Varoczy L., Papp G. (2009). Aspects of B-cell non-hodgkin's lymphoma development: a transition from immune-reactivity to malignancy. *Scandinavian Journal of Immunology*.

[4] Papp G., Szabó K., Szekanecz Z., Zeher M. (2014). Follicular helper T cells in autoimmune diseases. *Rheumatology*.

[5] Szabo K., Papp G., Dezso B., Zeher M. (2014). The histopathology of labial salivary glands in primary Sjögren's syndrome: focusing on follicular helper T cells in the inflammatory infiltrates. *Mediators of Inflammation*.

[6] Szabo K., Papp G., Barath S., Gyimesi E., Szanto A., Zeher M. (2013). Follicular helper T cells may play an important role in the severity of primary Sjögren's syndrome. *Clinical Immunology*.

[7] Kwok S. K., Lee J., Yu D. (2015). A pathogenetic role for IL-21 in primary Sjögren syndrome. *Nature Reviews Rheumatology*.

[8] Lindner S., Dahlke K., Sontheimer K. (2013). Interleukin 21-induced granzyme B-expressing B cells infiltrate tumors and regulate T cells. *Cancer Research*.

[9] Hagn M., Ebel V., Sontheimer K. (2010). CD5^+^ B cells from individuals with systemic lupus erythematosus express granzyme B. *European Journal of Immunology*.

[10] Vitali C., Bombardieri S., Jonsson R. (2002). Classification criteria for Sjögren's syndrome: a revised version of the European criteria proposed by the American-European Consensus Group. *Annals of the Rheumatic Diseases*.

[11] Jin H., Carrio R., Yu A., Malek T. R. (2004). Distinct activation signals determine whether IL-21 induces B cell costimulation, growth arrest, or Bim-dependent apoptosis. *The Journal of Immunology*.

[12] Hagn M., Sontheimer K., Dahlke K. (2012). Human B cells differentiate into granzyme B-secreting cytotoxic B lymphocytes upon incomplete T-cell help. *Immunology and Cell Biology*.

[13] Romero V., Andrade F. (2008). Non-apoptotic functions of granzymes. *Tissue Antigens*.

[14] Wensink A. C., Hack C. E., Bovenschen N. (2015). Granzymes regulate proinflammatory cytokine responses. *The Journal of Immunology*.

[15] Hagn M., Jahrsdörfer B. (2012). Why do human B cells secrete granzyme B? Insights into a novel B-cell differentiation pathway. *OncoImmunology*.

[16] Kroese F. G. M., Abdulahad W. H., Haacke E., Bos N. A., Vissink A., Bootsma H. (2014). B-cell hyperactivity in primary Sjögren's syndrome. *Expert Review of Clinical Immunology*.

